# Mitohormesis linking metabolic dysfunction and neurodegeneration: implications for dementia and therapeutic strategies

**DOI:** 10.3389/fnmol.2026.1892506

**Published:** 2026-09-15

**Authors:** Toshiki Otoda, Hideto Nakajima, Tadateru Takayama

**Affiliations:** 1 Division of General Medicine, Department of Internal Medicine, Nihon University School of Medicine, Itabashi-ku, Tokyo, Japan; 2 Division of Neurology, Department of Internal Medicine, Nihon University School of Medicine, Itabashi-ku, Tokyo, Japan

**Keywords:** dementia, metabolic disease, mitochondrial dysfunction, mitochondrial quality control, mitohormesis, neurodegeneration, oxidative stress, redox signaling

## Abstract

Mitochondria are central regulators of cellular metabolism, redox homeostasis, and stress adaptation. Mitohormesis refers to an adaptive response in which mild or transient mitochondrial perturbation activates stress-response pathways that subsequently enhance mitochondrial or cellular resilience; however, persistent or excessive stress can overwhelm adaptive capacity and promote mitochondrial dysfunction and tissue injury. Metabolic diseases, including obesity and type 2 diabetes mellitus, are major risk factors for cognitive decline and dementia, and clinical studies have demonstrated associations between metabolic dysfunction, structural brain abnormalities, accelerated brain aging, and impaired cognitive function. However, direct evidence linking mitochondrial dysfunction to neurodegeneration in humans remains limited, with most mechanistic insights derived from experimental animal models and cultured neuronal systems. Experimental evidence indicates that chronic metabolic stress can disrupt mitochondrial quality control and proteostasis, increase mitochondrial reactive oxygen species production, and promote neuroinflammation and neuronal dysfunction. Conversely, adaptive mitochondrial stress responses can preserve mitochondrial integrity and cellular resilience through coordinated regulation of the integrated stress response, mitochondrial quality-control mechanisms, lysosomal–mitochondrial crosstalk, extracellular vesicle-mediated communication, and inter-organ signaling. In Alzheimer’s disease, mitochondrial dysfunction and amyloid-β/tau pathology may interact bidirectionally, potentially generating self-reinforcing cycles of neuronal injury. Lifestyle and pharmacological interventions—including exercise; caloric restriction; nutritional ketosis; and the use of metformin, sodium–glucose cotransporter 2 inhibitors, and glucagon-like peptide-1 receptor agonists—have been associated with adaptive mitochondrial and metabolic responses involving AMP-activated protein kinase, nuclear factor erythroid 2-related factor 2, mitochondrial biogenesis, mitophagy, and redox signaling. However, evidence that mitohormesis directly mediates their beneficial effects varies substantially across interventions and remains predominantly indirect or hypothesized in humans. Moreover, the discrepancy between encouraging preclinical findings and clinical outcomes highlights important translational barriers, including the lack of validated biomarkers, uncertainty regarding optimal stress intensity and timing, and tissue- and disease-specific differences in adaptive capacity. Collectively, current evidence supports mitohormesis as a conceptual framework for integrating mitochondrial stress adaptation, metabolic dysfunction, and neuronal resilience rather than as an established unifying mechanism underlying neurodegeneration. Defining the conditions under which mitochondrial stress is adaptive, identifying reliable biomarkers of mitohormesis, and determining whether these responses can be safely and effectively modulated in humans will be essential for establishing its therapeutic relevance in metabolic and neurodegenerative diseases.

## 1 Introduction

Mitochondria are essential organelles that regulate cellular energy metabolism, redox homeostasis, and stress adaptation (Raafat et al., 2026). In highly energy-dependent tissues, such as the brain, heart, and kidney, mitochondrial integrity is critical for maintaining cellular function and survival. Beyond their classical role in energy production, mitochondria are increasingly recognized as dynamic signaling hubs that shape neuronal function, cellular stress responses, and cognitive processes. Mitochondrial dysfunction is a central pathological feature of both metabolic diseases, including type 2 diabetes mellitus and obesity, and neurodegenerative disorders, such as Alzheimer’s disease (AD) and related dementias (Ristow, 2014). In metabolic diseases, mitochondrial dysfunction has been documented in peripheral insulin-responsive tissues, including skeletal muscle, liver, adipose tissue, pancreatic β-cells, and cardiac and vascular tissues (Lowell and Shulman, 2005; Szendroedi et al., 2011), whereas in neurodegenerative disorders, it primarily affects neurons and glial cells within the central nervous system (Kann and Kovács, 2007; Tönnies and Trushina, 2017). These conditions share common pathogenic mechanisms, including impaired mitochondrial bioenergetics, oxidative stress, and dysregulated cellular stress responses (Ristow, 2014).

Although AD is the most extensively studied neurodegenerative cause of dementia and mechanistic evidence from AD-focused studies constitutes a substantial proportion of the evidence discussed in this review, dementia is a heterogeneous clinical syndrome encompassing AD, vascular dementia, Lewy body dementia, and mixed pathologies. Accordingly, mechanistic findings derived specifically from AD models are distinguished from evidence pertaining to dementia more broadly and should not be generalized to all forms of dementia.

Reactive oxygen species (ROS), produced primarily during mitochondrial oxidative phosphorylation (OXPHOS), have long been regarded as harmful byproducts that contribute to oxidative damage and disease progression (Mattson, 2008; Ristow and Schmeisser, 2014). However, accumulating evidence has substantially revised this view. Moderate and transient increases in mitochondrial ROS can activate adaptive stress-response pathways that enhance cellular resilience, improve mitochondrial function, and promote survival (Mattson, 2008; Kim et al., 2013; Wei et al., 2025). This adaptive phenomenon, termed mitohormesis, refers to a process in which mild mitochondrial stress induces compensatory responses that reprogram cellular metabolism, proteostasis, and redox homeostasis, thereby enhancing stress resistance (Da et al., 2024; Wei et al., 2025). Mitohormesis is highly context- and dose-dependent (Kang et al., 2021). Experimental studies in proopiomelanocortin (POMC) neuron-specific Crif1-deficient mouse models reported that mild mitoribosomal stress enhanced energy expenditure, thermogenesis, and resistance to diet-induced obesity, whereas severe mitochondrial stress impaired POMC neuronal function and promoted obesity and glucose intolerance. These studies reported the evaluation of systemic metabolic phenotypes, including body weight, energy expenditure, glucose homeostasis, adipose tissue thermogenesis, adipose tissue browning, and mitochondrial stress responses (Kang et al., 2021). These findings suggest that mitochondrial stress responses exert both adaptive and maladaptive effects depending on the intensity and duration of mitochondrial stress (Cunnane et al., 2020; Kang et al., 2021).

Emerging evidence from experimental animal models and human studies indicates that mitochondrial stress responses are not limited to cell-autonomous adaptation but also involve inter-organ communication mediated by stress-induced endocrine factors and extracellular signaling pathways (Coll et al., 2020; Gunawan et al., 2025). Mitokines such as fibroblast growth factor 21 (FGF21) and growth differentiation factor 15 (GDF15) have been identified as endocrine mediators of mitochondrial stress that contribute to systemic metabolic adaptation and help maintain metabolic homeostasis in both experimental models and human studies (Lin and Beal, 2006; Coll et al., 2020; Gunawan et al., 2025). Recent conceptual frameworks propose that neurodegenerative disorders may be understood, at least in part, as systemic diseases driven by impaired mitochondrial biology and disrupted stress adaptation (Shpilka and Haynes, 2018; Swerdlow, 2018). In this context, classical pathological features such as amyloid deposition and neuroinflammation may represent downstream manifestations of broader metabolic and organellar dysfunction.

The molecular mechanisms underlying mitohormesis, including the integrated stress response (ISR) and mitochondrial unfolded protein response (UPR*^mt^*), have been increasingly characterized through experimental studies in mammalian cell lines, primary cells, and genetically engineered mouse models, with additional support from translational and clinical studies (Pakos-Zebrucka et al., 2016; Cuadrado et al., 2019). Adaptive mitochondrial stress signaling involves the coordinated activation of redox-sensitive pathways, including nuclear factor erythroid 2-related factor 2 (NRF2), AMP-activated protein kinase (AMPK), mitochondrial biogenesis pathways, and mitochondrial quality-control mechanisms such as mitophagy (Mattson, 2008; Pickles et al., 2018; Pereira and Keipert, 2024). Through these responses, cells adapt to metabolic and oxidative stress while preserving mitochondrial and tissue integrity.

The concept of mitohormesis is particularly relevant to metabolic disease. Chronic metabolic stress associated with hyperglycemia, insulin resistance, and lipid overload exerts sustained pressure on mitochondria, disrupting redox homeostasis and impairing mitochondrial function. Although prolonged metabolic stress ultimately promotes mitochondrial dysfunction and tissue injury, adaptive mitochondrial responses may initially confer protection (Lin and Beal, 2006).

Mitochondrial dysfunction has been implicated in major causes of dementia, including AD and vascular cognitive impairment and dementia (Kann and Kovács, 2007). Neurons are highly dependent on mitochondrial OXPHOS to sustain synaptic transmission and neuronal maintenance (Tönnies and Trushina, 2017). Impaired mitochondrial function disrupts neuronal energy metabolism, increases oxidative stress, and promotes neuroinflammation, thereby contributing to neurodegeneration and cognitive decline (Cheng et al., 2023). Notably, metabolic diseases and neurodegenerative disorders are closely interconnected. Epidemiological and clinical studies have reported that type 2 diabetes and metabolic syndrome significantly increase the risk of cognitive impairment and dementia (de la Monte et al., 2016). Shared mechanisms, including mitochondrial dysfunction, oxidative stress, and chronic inflammation, link metabolic dysfunction to neurodegeneration (Sims-Robinson et al., 2010; De Haes et al., 2014). Collectively, these observations suggest that mitochondrial stress responses and mitohormetic mechanisms may represent common adaptive pathways that influence neuronal vulnerability and resilience across multiple organ systems, including the brain (Zuo et al., 2024).

Previous reviews have established the broader conceptual and mechanistic foundations of mitohormesis, including mitochondrial stress signaling, metabolic rewiring, proteostasis, and its potential relevance to neurodegenerative disorders (Cheng et al., 2023; Phillips and Picard, 2024; Benarroch, 2026). Building on these frameworks, the present review focuses specifically on the interface between systemic metabolic dysfunction and neuronal vulnerability and extends previous perspectives by integrating mitochondrial stress adaptation across multiple biological scales, including lysosomal–mitochondrial crosstalk, extracellular vesicle and mitokine signaling, and inter-organ communication. In addition, we apply explicit operational criteria to distinguish direct, indirect, and hypothesized evidence for mitohormesis across lifestyle and pharmacological interventions and critically evaluate the translational gap between experimental mitochondrial adaptation and clinical dementia outcomes. Thus, rather than proposing mitohormesis as a new concept, this review provides an evidence-stratified framework for evaluating its potential role at the intersection of metabolic disease and neurodegeneration.

Although mitohormesis is increasingly recognized as a potentially protective mechanism, its role in linking metabolic dysfunction and neurodegeneration remains incompletely understood (Zuo et al., 2024). In this review, we integrate current mechanistic and clinical evidence on mitochondrial stress adaptation across metabolic and neurodegenerative diseases, with particular emphasis on the distinction between adaptive mitochondrial stress responses and mitohormesis. We further examine how intracellular signaling, mitochondrial quality control, organelle crosstalk, and neural and humoral inter-organ communication may influence the balance between neuronal resilience and progressive dysfunction.

Importantly, throughout this review, mitohormesis is distinguished from a general mitochondrial stress response, mitochondrial adaptation, or mitochondrial protection. We consider evidence supportive of mitohormesis when a defined mitochondrial stressor induces a transient or moderate mitochondrial perturbation, activates adaptive stress-response pathways, and subsequently produces a demonstrable increase in mitochondrial or cellular stress resistance or resilience. Stronger evidence is provided when inhibition or genetic disruption of the relevant adaptive stress-response pathway attenuates or abolishes the beneficial phenotype. Conversely, activation of AMPK, NRF2, mitochondrial biogenesis, mitophagy, or antioxidant responses alone is considered insufficient to establish mitohormesis in the absence of evidence linking the initial mitochondrial perturbation to a subsequent adaptive benefit. Accordingly, evidence discussed in this review is categorized, where appropriate, as direct evidence of mitohormesis, indirect evidence compatible with mitohormesis, or a hypothesized mitohormetic mechanism.

### 1.1 Literature scope

This article is a narrative review and was not conducted according to a prospectively defined systematic or scoping review protocol. The literature discussed was selected to provide representative mechanistic, preclinical, translational, and clinical evidence relevant to mitochondrial stress adaptation, metabolic dysfunction, and neurodegeneration. Accordingly, the cited studies should not be interpreted to represent an exhaustive or systematic literature search.

## 2 Metabolic dysfunction and brain vulnerability

Metabolic diseases, including obesity and type 2 diabetes mellitus, are major risk factors for cognitive decline and dementia. These conditions expose multiple organs to chronic metabolic stress arising from hyperglycemia, insulin resistance, lipid overload, and adipose tissue dysfunction. In peripheral insulin-responsive tissues, including the liver, skeletal muscle, adipose tissue, pancreatic β-cells, and the cardiovascular system, sustained metabolic stress promotes mitochondrial dysfunction, oxidative stress, and inflammatory signaling. These peripheral disturbances can influence the brain through interconnected metabolic, inflammatory, and vascular pathways, including systemic insulin resistance, lipid overload, circulating inflammatory mediators, endothelial dysfunction, and impaired cerebrovascular homeostasis, thereby exposing neurons and glial cells to sustained metabolic and oxidative stress (Maciejczyk et al., 2019; Sanchez-Alegria and Arias, 2023; Wang et al., 2019).

At the cellular level, sustained metabolic stress can impair neuronal and glial mitochondrial bioenergetics, increase mitochondrial ROS production, and disrupt mitochondrial quality-control mechanisms, including mitochondrial dynamics and mitophagy. When these disturbances persist or exceed adaptive capacity, impaired mitochondrial quality control further amplifies oxidative stress and inflammatory signaling, promoting neuroinflammation, synaptic dysfunction, and progressive neuronal vulnerability (Heneka et al., 2015). Thus, a sequential mechanistic framework can be proposed in which systemic metabolic dysfunction promotes peripheral metabolic, inflammatory, and vascular disturbances, which in turn increase mitochondrial stress in neurons and glial cells; failure of mitochondrial stress adaptation then leads to impaired mitochondrial quality control, oxidative stress, neuroinflammation, and ultimately neuronal dysfunction and cognitive decline.

Clinical studies using structural brain magnetic resonance imaging (MRI) have reported that visceral adiposity is associated with impaired cognitive performance and structural brain abnormalities, including white matter lesions and enlarged perivascular spaces, suggesting that metabolic dysregulation adversely affects brain integrity (Ozato et al., 2021). Recent systematic reviews further indicate that central obesity and metabolic syndrome are associated with reduced total brain volume, gray matter atrophy, an increased burden of white matter hyperintensities, and impaired white matter integrity assessed via diffusion tensor imaging, supporting a widespread impact of metabolic dysfunction on brain structure (Soleymani et al., 2025). Beyond obesity itself, qualitative features of body composition also contribute to risk. Sarcopenic obesity, defined by the coexistence of increased fat mass and reduced skeletal muscle mass, has emerged as a particularly high-risk condition associated with markedly increased risks of cognitive impairment and dementia (Someya et al., 2022; Tang et al., 2025). Longitudinal cohort studies in Asian populations have also reported that both midlife obesity and subsequent weight loss are associated with an increased risk of dementia. Weight loss confers an even greater risk than weight gain, suggesting that dynamic metabolic stress may contribute to neurodegeneration (Tashiro et al., 2023).

Type 2 diabetes mellitus further exacerbates this association. Large-scale human neuroimaging studies combining structural MRI, functional MRI, cognitive assessment, and meta-analytic data have reported that individuals with type 2 diabetes exhibit accelerated brain aging, characterized by gray matter atrophy, particularly in the ventral striatum, cerebellum, and putamen, together with functional brain reorganization and progressive cognitive decline. Longer disease duration is associated with more pronounced structural and functional brain abnormalities, suggesting that chronic metabolic stress may accelerate age-related brain changes (Antal et al., 2022). More recent evidence extends these observations by indicating that even prediabetes is associated with increased brain age and that this relationship can be significantly attenuated by healthy lifestyle behaviors (Dove et al., 2024). Consistent with these findings, large-scale neuroimaging analyses have further demonstrated that modifiable metabolic and vascular risk factors preferentially affect brain regions that are particularly vulnerable to aging and neurodegenerative diseases (Manuello et al., 2024).

Although these neuroimaging findings strongly support an association between metabolic disease and structural brain abnormalities, direct evidence linking systemic metabolic dysfunction to neuronal mitochondrial damage in humans remains limited. Nevertheless, recent systematic evidence from human postmortem brain tissue and human-derived cellular models indicates that AD is associated with alterations in mitochondrial enzymes involved in carbon entry into the tricarboxylic acid cycle, oxidative phosphorylation, and redox regulation, with particularly consistent evidence for dysfunction of pyruvate dehydrogenase and α-ketoglutarate dehydrogenase complexes (Asadi Anar et al., 2026). Most evidence defining the sequential mechanisms by which systemic metabolic stress disrupts neuronal mitochondrial homeostasis continues to be derived from experimental animal models and cultured neuronal systems.

Experimental studies in high-fat diet-fed mice have reported that metabolic overload induces mitochondrial oxidative stress and neuroinflammation, whereas studies in amyloid precursor protein/presenilin 1 (APP/PS1) transgenic mice have shown that impaired mitochondrial quality control is associated with accelerated amyloid pathology and cognitive decline. Similarly, studies in diabetic mouse models and cultured neuronal cells have reported that chronic metabolic stress disrupts mitochondrial quality control, increases mitochondrial ROS production, impairs mitophagy, and promotes neuroinflammation. Collectively, these findings support a model in which chronic metabolic stress progressively challenges adaptive mitochondrial stress responses and, when adaptive capacity becomes insufficient, increases neuronal vulnerability and promotes neurodegenerative processes. These observations are consistent with the broader framework in which mitochondrial dysfunction is recognized as a hallmark of aging and an important contributor to age-related disease (López-Otín et al., 2023). The proposed mechanistic sequence linking metabolic dysfunction, impaired mitochondrial stress adaptation, mitochondrial quality-control failure, neuroinflammation, and neurodegeneration is summarized in Figure 1 and discussed in detail in the following sections.

**Figure 1:**
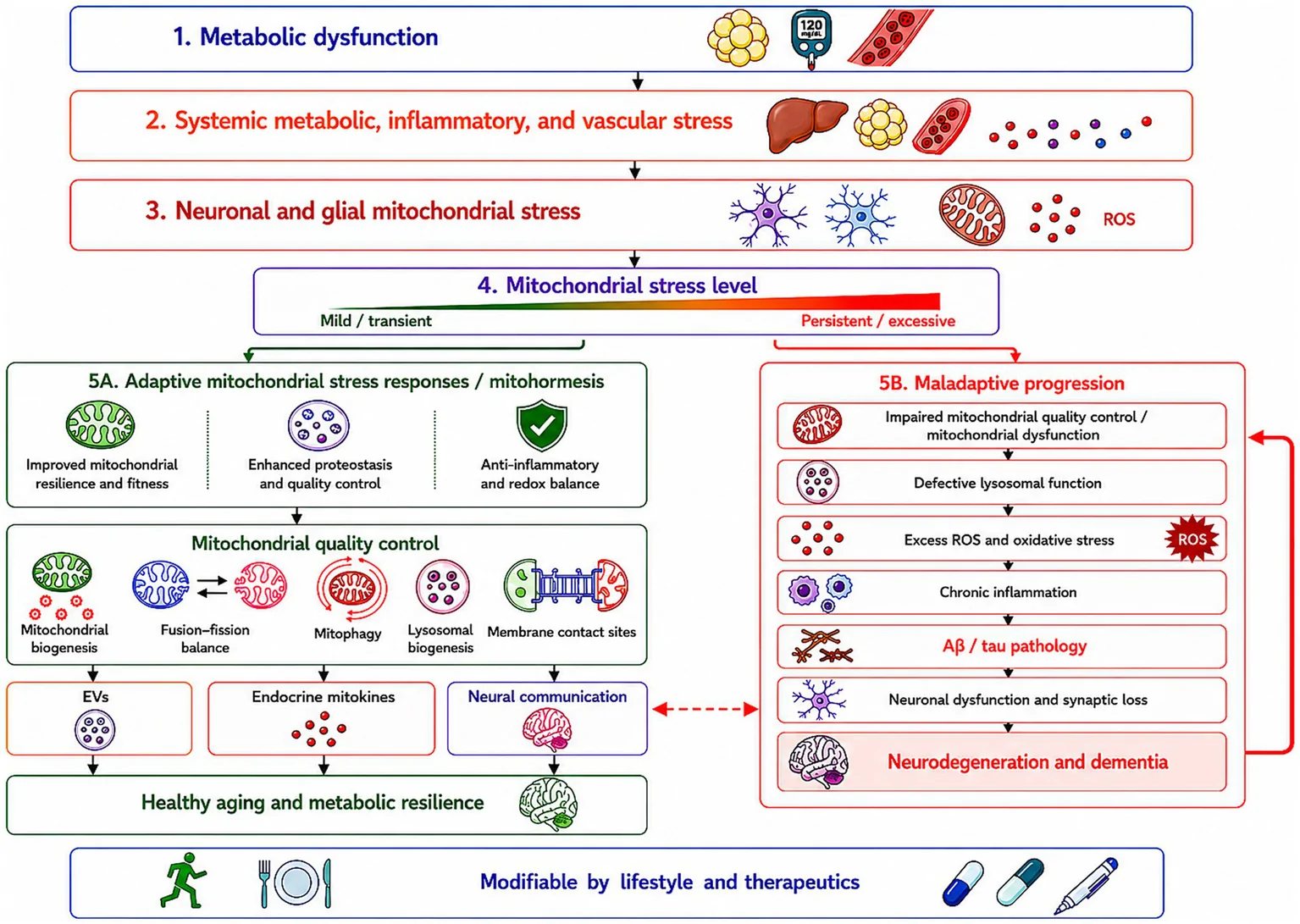
Proposed framework linking metabolic dysfunction, mitochondrial stress adaptation, and neurodegeneration. Metabolic dysfunction associated with obesity, type 2 diabetes mellitus, insulin resistance, dyslipidemia, and related metabolic and vascular abnormalities generates systemic metabolic, inflammatory, and vascular stress that can increase mitochondrial stress in neurons and glial cells. The biological consequences of mitochondrial stress depend on its intensity, duration, and the capacity of adaptive stress responses. Mild or transient mitochondrial perturbations may engage adaptive mitochondrial stress responses, including mitochondrial biogenesis, fusion–fission dynamics, mitophagy, proteostasis, and lysosomal biogenesis. Lysosomal–mitochondrial crosstalk, including VAMP7–Rab7-dependent membrane contact sites, and systemic communication through extracellular vesicles, endocrine mitokines, and neural pathways may further coordinate mitochondrial quality control and metabolic adaptation, thereby supporting metabolic and neuronal resilience. In contrast, persistent or excessive mitochondrial stress can overwhelm adaptive capacity, leading to impaired mitochondrial and lysosomal quality control, excessive oxidative stress, chronic inflammation, and neuronal dysfunction. In Alzheimer’s disease, Amyloid-β and Tau pathology may further amplify mitochondrial dysfunction through bidirectional interactions, contributing to a self-reinforcing cycle of neuronal injury. Lifestyle and pharmacological interventions may modulate these stress-response pathways, although the extent to which their effects are directly mediated by mitohormesis remains intervention- and evidence-dependent. Aβ, amyloid-β; EVs, extracellular vesicles; ROS, reactive oxygen species.

## 3 Molecular mechanisms of mitohormesis

### 3.1 ROS-dependent adaptive signaling

Mitohormesis is mediated by a coordinated network of mitochondrial stress-responsive signaling pathways that translate mild mitochondrial perturbations into adaptive cellular responses (De Haes et al., 2014; Ristow and Schmeisser, 2014). Rather than functioning solely as sources of oxidative damage, mitochondria act as dynamic signaling hubs that integrate metabolic and stress responses (Gunawan et al., 2025). Central to this process is the concept that moderate increases in mitochondrial ROS serve as signaling molecules. These ROS signals activate protective pathways and enhance cellular resilience.

Experimental studies in *Caenorhabditis elegans* (*C. elegans*), together with mitochondrial functional assays and isolated mitochondrial preparations, reported that mild mitochondrial stress elicits adaptive ROS signaling that promotes stress resistance and metabolic adaptation. For example, metformin partially inhibited mitochondrial complex I, resulting in transient mitochondrial ROS production and activation of adaptive stress-response pathways (De Haes et al., 2014). Notably, pharmacological scavenging of ROS using antioxidants, including N-acetylcysteine and butylated hydroxyanisole, abolished many of these beneficial effects, supporting the concept that controlled oxidative stress is required for mitohormetic adaptation (De Haes et al., 2014).

Mechanistic studies in *C. elegans*, together with isolated mitochondria and biochemical analyses, reported that redox-sensitive signaling mediators, including peroxiredoxins, serve as key sensors and transducers of oxidative stress signals (De Haes et al., 2014). Peroxiredoxins are thiol-dependent antioxidant enzymes that detoxify hydrogen peroxide and function as redox relay proteins, transmitting oxidative signals to downstream stress-response pathways. By coordinating the activation of these pathways, peroxiredoxins couple mitochondrial ROS signaling to adaptive transcriptional responses and cellular defense mechanisms, thereby linking mitochondrial stress to cellular resilience.

### 3.2 AMPK–NRF2 signaling and metabolic adaptation

Experimental studies in cultured mammalian cells, genetically engineered mouse models, and neuronal systems have reported AMPK as a central regulator of mitohormesis by integrating cellular energy status with adaptive stress responses (Hardie, 2008). Mild energetic stress resulting from altered mitochondrial function increases the AMP/ adenosine triphosphate (ATP) ratio, leading to AMPK activation, which promotes mitochondrial biogenesis through activation of peroxisome proliferator-activated receptor γ coactivator-1α (PGC-1α), a master regulator of this process (Jäger et al., 2007). Activated PGC-1α further induces nuclear respiratory factor 1 (NRF1), a transcription factor that regulates nuclear-encoded mitochondrial genes, and mitochondrial transcription factor A (TFAM), which controls mitochondrial DNA (mtDNA) transcription and replication, thereby coordinating mitochondrial biogenesis (Scarpulla, 2008). In addition, AMPK enhances fatty acid oxidation and suppresses energy-consuming anabolic pathways, thereby improving mitochondrial stress resistance.

Experimental studies in mammalian cells, mouse models, and neuronal systems have reported that the redox-responsive transcription factor NRF2, a master regulator of cellular antioxidant defense, orchestrates antioxidant defense programs and mitochondrial quality-control pathways (Yamamoto et al., 2018; Kasai et al., 2020). Upon mild oxidative stress, NRF2 dissociates from KEAP1, translocates to the nucleus, and induces the expression of antioxidant enzymes, including heme oxygenase-1, NAD(P)H quinone oxidoreductase 1, glutathione peroxidases, catalase, superoxide dismutases (SODs), and peroxiredoxins, thereby restoring redox homeostasis and limiting excessive ROS accumulation (Ma, 2013; Tonelli et al., 2018).

Peroxiredoxins are thiol-dependent antioxidant enzymes that detoxify hydrogen peroxide and function as redox relay proteins. Upon oxidation of their catalytic cysteine residues by hydrogen peroxide, peroxiredoxins undergo reversible conformational changes and transfer oxidative equivalents to target proteins through thiol-disulfide exchange reactions, thereby activating downstream stress-response signaling pathways (Rhee and Woo, 2011). Consequently, ROS signaling serves as the upstream trigger, AMPK functions as the metabolic integrator, and NRF2 acts as the transcriptional executor that coordinates antioxidant defense and mitochondrial quality control. Together, these interconnected pathways establish a robust adaptive network that enhances mitochondrial function, metabolic flexibility, and cellular resilience under conditions of mild mitochondrial stress.

### 3.3 ISR and mitochondrial proteostasis

Mitochondrial proteostasis refers to the maintenance of mitochondrial protein homeostasis through the coordinated regulation of protein import, folding, quality control, and degradation. Mitochondrial proteotoxic stress arises when unfolded or misfolded proteins accumulate within mitochondria or when mitochondrial protein import is impaired, thereby disrupting mitochondrial proteostasis and triggering adaptive stress-response pathways.

Experimental studies in cultured mammalian cells, including HAP1 and HEK293T cells, together with CRISPR-based genetic screens, have reported that mitochondrial proteotoxic stress activates the ISR through the overlapping activity with m-AAA protease 1 (OMA1)–DELE1– heme-regulated inhibitor kinase (HRI) signaling axis (Fessler et al., 2020; Guo et al., 2020). Activation of the inner mitochondrial membrane protease OMA1 results in the cleavage of DELE1, allowing the cleaved protein to accumulate in the cytosol, where it activates HRI, leading to phosphorylation of eIF2α and preferential translation of activating transcription factor 4 (ATF4). This signaling cascade transiently suppresses global protein synthesis while selectively inducing genes involved in antioxidant defense, amino acid metabolism, proteostasis, and mitochondrial adaptation.

ISR signaling is context-dependent because its downstream effects depend on the severity and duration of mitochondrial stress. Under mild or reversible stress, the ISR promotes mitochondrial recovery by transiently suppressing global protein synthesis while enhancing mitochondrial protein quality control, antioxidant defense, amino acid metabolism, and restoration of mitochondrial function. In contrast, under persistent or severe mitochondrial damage, mitochondrial protein import becomes markedly impaired, resulting in PTEN-induced kinase 1 (PINK1) stabilization and activation of mitophagy to eliminate irreversibly damaged mitochondria (Yang et al., 2026).

The UPR*^mt^* constitutes another complementary adaptive pathway that preserves mitochondrial proteostasis. Experimental evidence from mammalian cells and animal models has shown that UPR*^mt^* is activated by the accumulation of unfolded or misfolded mitochondrial proteins and induces mitochondrial chaperones and proteases, including HSP60, mtHSP70, and ClpP, thereby promoting protein refolding and degradation of damaged proteins (Jovaisaite et al., 2014; Shpilka and Haynes, 2018). Although ISR and UPR*^mt^* are activated through distinct molecular mechanisms, increasing evidence suggests that they are functionally interconnected. ATF4 regulates adaptive metabolic and proteostatic programs and contributes to activating transcription factor 5-dependent UPR*^mt^* transcriptional responses, thereby promoting expression of mitochondrial chaperones and proteases that restore mitochondrial protein homeostasis (Fiorese et al., 2016; Shpilka and Haynes, 2018). Together, these pathways converge to reinforce mitochondrial proteostasis, maintain bioenergetic function, and enhance cellular resilience during metabolic and oxidative stress.

### 3.4 Mitochondrial quality control and organelle communication

Beyond redox signaling, mitochondrial quality-control mechanisms are essential components of mitohormesis. Mitochondrial quality control integrates mitochondrial biogenesis, fusion–fission dynamics, mitophagy, and proteostasis to preserve a functional mitochondrial network under both physiological and stress conditions. Mitochondrial biogenesis replenishes healthy mitochondria and maintains bioenergetic capacity, whereas mitophagy selectively removes dysfunctional mitochondria, thereby limiting excessive ROS accumulation and preventing the propagation of mitochondrial damage (Kim et al., 2013; Wang Q. et al., 2026). Together, these coordinated processes maintain mitochondrial homeostasis and sustain adaptive mitohormetic responses. Experimental studies in mammalian cells, mouse models, and neuronal systems have reported that mitochondrial dynamics are tightly coordinated with mitochondrial quality-control mechanisms (Youle and van der Bliek, 2012; Pickles et al., 2018). Mitochondrial fusion is mediated primarily by the outer mitochondrial membrane GTPases mitofusin-1 and mitofusin-2, together with the inner mitochondrial membrane GTPase optic atrophy protein 1, thereby promoting mitochondrial content mixing and functional complementation. Conversely, mitochondrial fission is regulated by the cytosolic GTPase dynamin-related protein 1, which is recruited to the outer mitochondrial membrane upon activation to facilitate the segregation of damaged mitochondrial fragments before mitophagy (Youle and van der Bliek, 2012). The PINK1–Parkin pathway represents one of the best-characterized mechanisms regulating stress-induced mitophagy (Youle and Narendra, 2011). Under physiological conditions, PINK1 is continuously imported into mitochondria and rapidly degraded. Loss of mitochondrial membrane potential prevents its import and degradation, resulting in the stabilization of PINK1 on the outer mitochondrial membrane, where it recruits and activates the cytosolic E3 ubiquitin ligase Parkin. Activated Parkin translocates to damaged mitochondria and ubiquitinates multiple outer mitochondrial membrane proteins, thereby promoting the selective lysosomal degradation of dysfunctional mitochondria through mitophagy. This pathway links mitochondrial dynamics with lysosomal quality control and preserves mitochondrial homeostasis under stress conditions (Pickles et al., 2018). Increasing evidence indicates that mitochondrial quality control is coordinated by mitochondria-autonomous mechanisms and through functional communication with lysosomes, which together regulate mitochondrial turnover, proteostasis, and adaptive stress responses.

Crosstalk between mitochondria and other organelles, particularly lysosomes, further links mitochondrial stress responses to autophagy–lysosomal pathways. Efficient lysosomal function is indispensable for the completion of mitophagy, whereas lysosomal dysfunction results in the accumulation of damaged mitochondria, persistent oxidative stress, and impaired proteostasis, thereby contributing to neurodegeneration (Menzies et al., 2017). Transcription factor EB, a master regulator of lysosomal biogenesis, coordinates lysosomal function with mitochondrial metabolism by promoting lysosomal biogenesis, autophagy, and organelle quality control, thereby facilitating adaptive mitochondrial stress responses (Lim and Zoncu, 2016). Thus, mitochondrial adaptation depends on the coordinated regulation of both mitochondrial and lysosomal quality-control systems. Recent reviews have emphasized that lysosomal stress is increasingly recognized as a common upstream mechanism linking impaired organelle quality control to oxidative stress, inflammation, and tissue injury across multiple chronic diseases (Otoda et al., 2025a). These observations support the lysosomal–mitochondrial axis theory, which proposes that impaired lysosomal degradation contributes to mitochondrial dysfunction, oxidative stress, and progressive cellular decline (Terman et al., 2006).

Recent studies further report that mitochondria–lysosome membrane contacts function as dynamic regulatory platforms that coordinate mitochondrial dynamics, organelle communication, and neuronal homeostasis. Disruption of *vesicle-associated membrane protein 7* (VAMP7)-dependent mitochondria–lysosome contacts impairs *Ras-related protein Rab7* (Rab7)-mediated regulation of mitochondrial dynamics, leading to oxidative stress and dopaminergic neurodegeneration, further emphasizing the importance of lysosomal–mitochondrial communication in maintaining neuronal resilience (Wang H. et al., 2026).

Experimental evidence further indicates that chronic metabolic stress disrupts mitochondrial function and lysosomal quality-control systems. In cultured cells and animal models, excessive free fatty acids induce lysosomal–mitochondrial dysfunction, thereby linking metabolic overload to mitochondrial dysfunction and oxidative stress (Li et al., 2008). More recent mechanistic studies have reported that saturated fatty acids impair lysosomal function through oxidative stress-induced perilysosomal Ca^2+^ overload and persistent mammalian target of rapamycin complex 1 (mTORC1) activation, thereby suppressing autophagic flux and promoting the accumulation of damaged cellular components (Nguyen et al., 2025). These findings suggest that chronic metabolic stress disrupts the coordinated function of mitochondrial and lysosomal quality-control systems, resulting in impaired mitochondrial adaptation, defective proteostasis, and increased susceptibility to tissue injury. Within the framework of mitohormesis, failure of this integrated organelle stress response may shift adaptive mitochondrial signaling toward maladaptive cellular dysfunction.

Beyond intracellular degradation pathways, extracellular vesicle (EV)-mediated export of mitochondrial components represents an additional mechanism of mitochondrial quality control and intercellular mitohormetic signaling. Experimental studies in cultured adipocytes and mouse models have reported that metabolically stressed adipocytes release EVs containing mitochondrial proteins, mtDNA, and other stress-associated cargo (Crewe et al., 2021; Kim and Lee, 2024). These EVs can be internalized by neighboring or distant recipient cells, including cardiac cells, where their mitochondrial cargo induces transient ROS signaling and adaptive stress responses. Thus, EV-mediated transfer enables stressed donor cells to communicate their mitochondrial status beyond the originating tissue and coordinate adaptive responses in recipient cells. This mechanism complements intracellular lysosomal degradation and classical endocrine signaling by extending mitochondrial quality control from a cell-autonomous process to an intercellular and inter-organ communication network (Crewe et al., 2021; Todkar et al., 2021; Picca et al., 2023; König and McBride, 2024; Hah et al., 2026).

Recent studies further report that mitochondrial protein import stress activates adaptive ISR signaling and coordinates mitochondrial quality control with cytosolic proteostasis, further supporting the concept that mitochondrial adaptation depends on integrated inter-organelle communication rather than isolated quality-control pathways (Wrobel et al., 2015; Shpilka and Haynes, 2018; Fessler et al., 2020). Collectively, these intracellular and intercellular communication pathways enable mitochondria to coordinate local quality-control mechanisms with systemic metabolic adaptation, thereby determining whether mitochondrial stress promotes adaptive resilience or maladaptive cellular dysfunction. Figure 2 summarizes the major molecular mechanisms underlying adaptive and maladaptive mitochondrial stress responses, including lysosomal–mitochondrial crosstalk.

**Figure 2:**
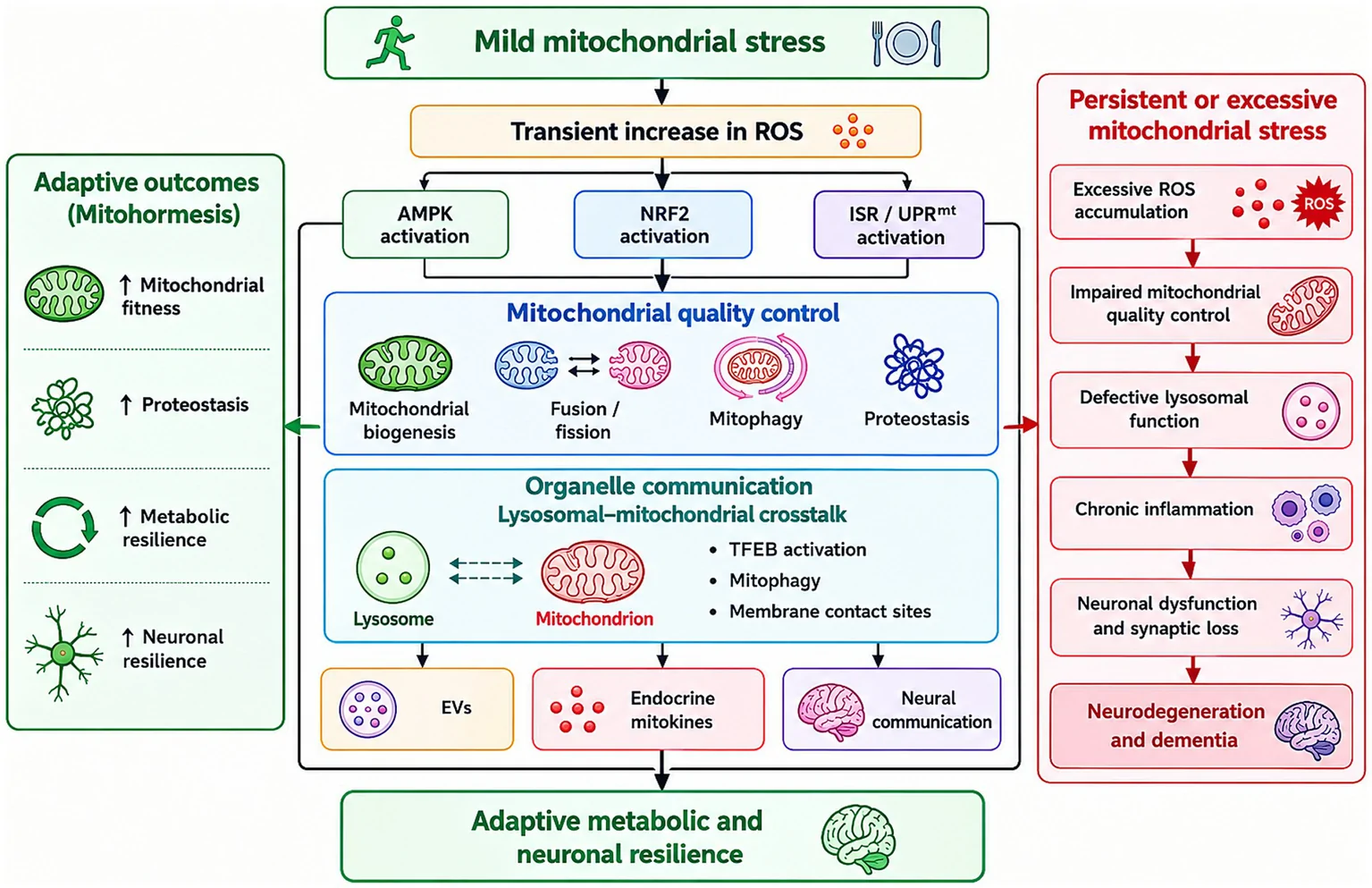
Molecular mechanisms underlying adaptive mitochondrial stress responses and the transition to maladaptive mitochondrial dysfunction. Mild or transient mitochondrial perturbation can generate redox and metabolic signals that activate adaptive stress-response pathways, including AMPK, NRF2, ISR, and UPR*^mt^*. These pathways coordinate mitochondrial biogenesis and dynamics, mitophagy, proteostasis, and other mitochondrial quality-control mechanisms that may enhance cellular resilience. When a defined mitochondrial perturbation elicits such adaptive responses and subsequently increases mitochondrial or cellular resilience, this response is consistent with mitohormesis. Mitochondrial quality control is further coordinated through organelle communication, particularly lysosomal–mitochondrial crosstalk involving TFEB-dependent lysosomal regulation, mitophagy, and mitochondria–lysosome membrane contact sites involving VAMP7 and Rab7. EVs, endocrine mitokines, and neural pathways may extend mitochondrial stress signaling beyond individual cells and contribute to systemic metabolic adaptation. In contrast, persistent or excessive mitochondrial stress can exceed adaptive capacity, leading to impaired mitochondrial quality control, defective lysosomal function, excessive oxidative stress, chronic inflammation, neuronal dysfunction, and ultimately neurodegeneration. Thus, activation of individual stress-response pathways alone should not be interpreted as sufficient evidence of mitohormesis without demonstration of a subsequent adaptive or resilience-enhancing phenotype. AMPK, AMP-activated protein kinase; EVs, extracellular vesicles; ISR, integrated stress response; NRF2, nuclear factor erythroid 2-related factor 2; Rab7, Ras-related protein Rab-7A; ROS, reactive oxygen species; TFEB, transcription factor EB; UPR*^mt^*, mitochondrial unfolded protein response; VAMP7, vesicle-associated membrane protein 7.

### 3.5 Inter-organ communication and systemic mitohormesis

Beyond intracellular signaling, mitohormetic responses also involve systemic inter-organ communication through endocrine and extracellular pathways. Experimental studies in cultured cells and animal models, together with complementary human studies, have reported the identification of stress-induced endocrine factors, termed mitokines, as key mediators that transmit mitochondrial stress signals to distant tissues (Burtscher et al., 2023; Ji et al., 2026). Among these, FGF21 and GDF15 are the best-characterized mammalian mitokines. FGF21 is released from metabolically active tissues, including the liver and skeletal muscle, in response to mitochondrial and metabolic stress, whereas GDF15 is induced in stressed tissues, including the liver and kidney, and functions as an endocrine mediator of mitochondrial stress (Katsumura et al., 2022; Burtscher et al., 2023).

FGF21 primarily regulates systemic energy metabolism by promoting fatty acid oxidation, insulin sensitivity, and metabolic flexibility, whereas GDF15 contributes to whole-body energy balance by regulating appetite and adaptive energy expenditure. Together, these endocrine mitokines extend mitochondrial stress responses beyond the originating tissue and coordinate organism-wide metabolic adaptation (Suomalainen and Battersby, 2018; Klaus and Ost, 2020; Burtscher et al., 2023). In addition to endocrine signaling, systemic mitohormesis may also be mediated by the EV-dependent transfer of mitochondrial stress signals, as described above.

Mitochondrial stress responses may also propagate through neural circuits. Experimental studies in genetically engineered mouse models reported that mild mitochondrial stress in hypothalamic POMC neurons activates the UPR*^mt^*, which subsequently enhances sympathetic nervous system activity and induces thermogenic adaptation in peripheral adipose tissue (Kang et al., 2021). This pathway provides a direct mechanism by which mitochondrial stress within the central nervous system is translated into coordinated peripheral metabolic adaptation through autonomic regulation. These findings indicate that neuronal networks complement endocrine and extracellular signaling pathways in coordinating whole-body metabolic adaptation.

Collectively, endocrine mitokines, EVs, and neural circuits represent complementary communication systems through which local mitochondrial stress is translated into coordinated organism-wide adaptive responses. These integrated pathways extend mitohormesis beyond individual cells and tissues, thereby maintaining systemic metabolic homeostasis and enhancing organismal resilience under physiological stress conditions (Klaus and Ost, 2020; Kim and Lee, 2024; Burtscher et al., 2023).

### 3.6 Adaptive resilience and maladaptive transition

Nonetheless, persistent mitochondrial dysfunction caused by prolonged hyperglycemia, lipid overload, chronic inflammation, aging, or neurodegenerative processes may overwhelm adaptive mitochondrial stress responses, resulting in sustained oxidative stress, impaired proteostasis, defective mitophagy, mtDNA damage, and progressive cellular dysfunction (Mattson, 2008; Da et al., 2024). Collectively, evidence from experimental studies in cultured cells, animal models, and human tissues indicates that ROS signaling, ISR activation, mitochondrial quality control, antioxidant defense, and inter-organelle communication function as an integrated adaptive network rather than as independent pathways. Under conditions of mild or transient mitochondrial stress, these coordinated responses preserve mitochondrial homeostasis, enhance metabolic flexibility, and strengthen cellular resilience in both metabolic and neurodegenerative disorders (Ristow and Schmeisser, 2014; Pakos-Zebrucka et al., 2016; Shpilka and Haynes, 2018; Gunawan et al., 2025). However, when mitochondrial stress becomes persistent or exceeds the capacity of these adaptive mechanisms, adaptive signaling is progressively replaced by excessive ROS accumulation, impaired ATP production, defective mitophagy, chronic inflammation, and irreversible cellular dysfunction, thereby contributing to the progression of metabolic disease and neurodegeneration (Mattson, 2008; Da et al., 2024).

## 4 Mitohormesis in metabolic diseases: linking metabolic dysfunction to cognitive decline and dementia risk

### 4.1 Modifiable risk factors and adaptive metabolic stress responses

Epidemiological evidence suggests that a substantial proportion of dementia cases are preventable through modification of risk factors, including physical inactivity, obesity, diabetes, and vascular conditions. Recent estimates indicate that up to 45% of dementia cases may be attributable to potentially modifiable risk factors, highlighting the importance of early and sustained preventive interventions (Livingston et al., 2024).

Multidomain intervention trials have further supported this concept. The Finnish Geriatric Intervention Study to Prevent Cognitive Impairment and Disability (FINGER), a randomized controlled trial involving 1,260 older adults, aged 60–77 years, who were at increased risk of dementia, demonstrated that a 2-year multidomain intervention consisting of dietary counseling, regular physical exercise, cognitive training, social activities, and intensive vascular risk management significantly improved or maintained cognitive performance compared with standard health advice (Ngandu et al., 2015). These findings indicate that coordinated lifestyle interventions can support cognitive health by simultaneously targeting multiple metabolic and vascular risk factors.

Experimental studies in rodent models complement these clinical observations by demonstrating that regular aerobic exercise enhances mitochondrial biogenesis through activation of the AMPK–PGC-1α–TFAM signaling pathway, improves mitochondrial quality control through mitophagy, reduces oxidative stress, and enhances hippocampal synaptic plasticity (Mattson, 2008; Ristow and Schmeisser, 2014; Pereira and Keipert, 2024; Gunawan et al., 2025). Dietary interventions, including caloric restriction and intermittent fasting, also engage nutrient- and energy-sensing pathways and can activate adaptive mitochondrial stress responses, whereas cognitive stimulation promotes neuronal plasticity and may support mitochondrial resilience. These interventions therefore engage biological pathways that overlap with adaptive mitochondrial stress responses; however, their beneficial effects should not be attributed to mitohormesis unless a defined mitochondrial perturbation is followed by an adaptive response and demonstrable enhancement of cellular or organismal resilience.

Consistent with this broader framework, population-based studies have identified multiple modifiable risk factors for dementia, including cardiometabolic disorders such as diabetes and cardiovascular disease, as well as chronic inflammatory states, including in younger populations (Hendriks et al., 2024). Furthermore, healthy lifestyle behaviors, including regular physical activity, a balanced diet, avoidance of smoking, and moderation of alcohol consumption, have been associated with attenuation of the relationship between metabolic disorders and accelerated brain aging.

### 4.2 Mitohormesis as a mechanistic bridge linking metabolic dysfunction to neurodegeneration

Experimental studies in neuronal cell cultures, genetically engineered mouse models, and complementary models of metabolic disease have reported that mitochondrial stress responses are not confined to peripheral metabolic organs but also play critical roles in regulating neuronal survival, synaptic function, and cognitive performance through mitochondrial quality-control pathways and proteostatic adaptation (Pereira and Keipert, 2024). These studies further report that impaired adaptive mitochondrial stress signaling promotes oxidative stress, defective mitophagy, neuroinflammation, and progressive neuronal dysfunction, thereby providing a mechanistic link between metabolic disorders and neurodegeneration.

Importantly, in AD, the relationship between mitochondrial dysfunction and Aβ/tau pathology is bidirectional rather than strictly linear. Although impaired mitochondrial quality control, oxidative stress, and defective proteostasis can promote the accumulation of amyloid-β (Aβ) and pathological tau, Aβ and tau pathology can in turn impair mitochondrial bioenergetics, dynamics, and axonal mitochondrial transport, thereby further increasing oxidative stress and neuronal vulnerability (Zhang et al., 2022). This reciprocal interaction may establish a self-reinforcing feed-forward cycle in which progressive neurodegenerative pathology further compromises mitochondrial stress adaptation and accelerates the transition from adaptive to maladaptive mitochondrial responses. Thus, metabolic dysfunction and impaired mitohormesis may increase susceptibility to neurodegeneration, while established AD pathology may subsequently amplify mitochondrial dysfunction.

Collectively, evidence from experimental models, together with clinical observations, supports the concept that mitohormesis functions as an integrative mechanism linking systemic metabolism to brain health (Pereira and Keipert, 2024). This concept is further supported by recent reviews identifying mitochondrial dysfunction as a central pathogenic mechanism and therapeutic target in Alzheimer’s disease (Moawad et al., 2025). The biological consequences of metabolic stress depend on its intensity, duration, and the capacity of adaptive mitochondrial stress responses. Whereas transient mitochondrial stress promotes mitochondrial resilience and metabolic adaptation, persistent or excessive mitochondrial stress results in oxidative damage, impaired mitochondrial quality control, and progressive neurodegeneration. These observations collectively support the concept that mitohormesis may serve as an adaptive hub integrating mitochondrial quality control, metabolic stress responses, and neurodegeneration. This concept is consistent with the expanding framework of the hallmarks of aging, in which mitochondrial dysfunction, impaired proteostasis, and chronic inflammation are recognized as interconnected drivers of age-related diseases (López-Otín et al., 2023).

## 5 Therapeutic modulation of mitochondrial stress responses

### 5.1 Conceptual framework for therapeutic modulation of adaptive mitochondrial stress responses

Therapeutic modulation of mitochondrial stress responses has emerged as a potential strategy for improving cellular resilience in metabolic and neurodegenerative diseases. Recent evidence further suggests that lysosomal–mitochondrial crosstalk represents an important target for preserving adaptive mitochondrial stress responses. Dysregulation of the lysosomal–mitochondrial axis has increasingly been implicated in cellular senescence and age-related dysfunction (Park et al., 2018). Because lysosomes regulate autophagy, mitophagy, and mitochondrial quality control, interventions targeting lysosomal function may influence the adaptive capacity of cells to mitochondrial stress and may help prevent the transition toward maladaptive mitochondrial dysfunction. Figure 3 and Table 1 summarize representative pharmacological and lifestyle interventions that modulate mitochondrial and metabolic stress-response pathways, together with their principal molecular targets, levels of evidence, strength of evidence for mitohormesis, and potential clinical implications.

**Figure 3:**
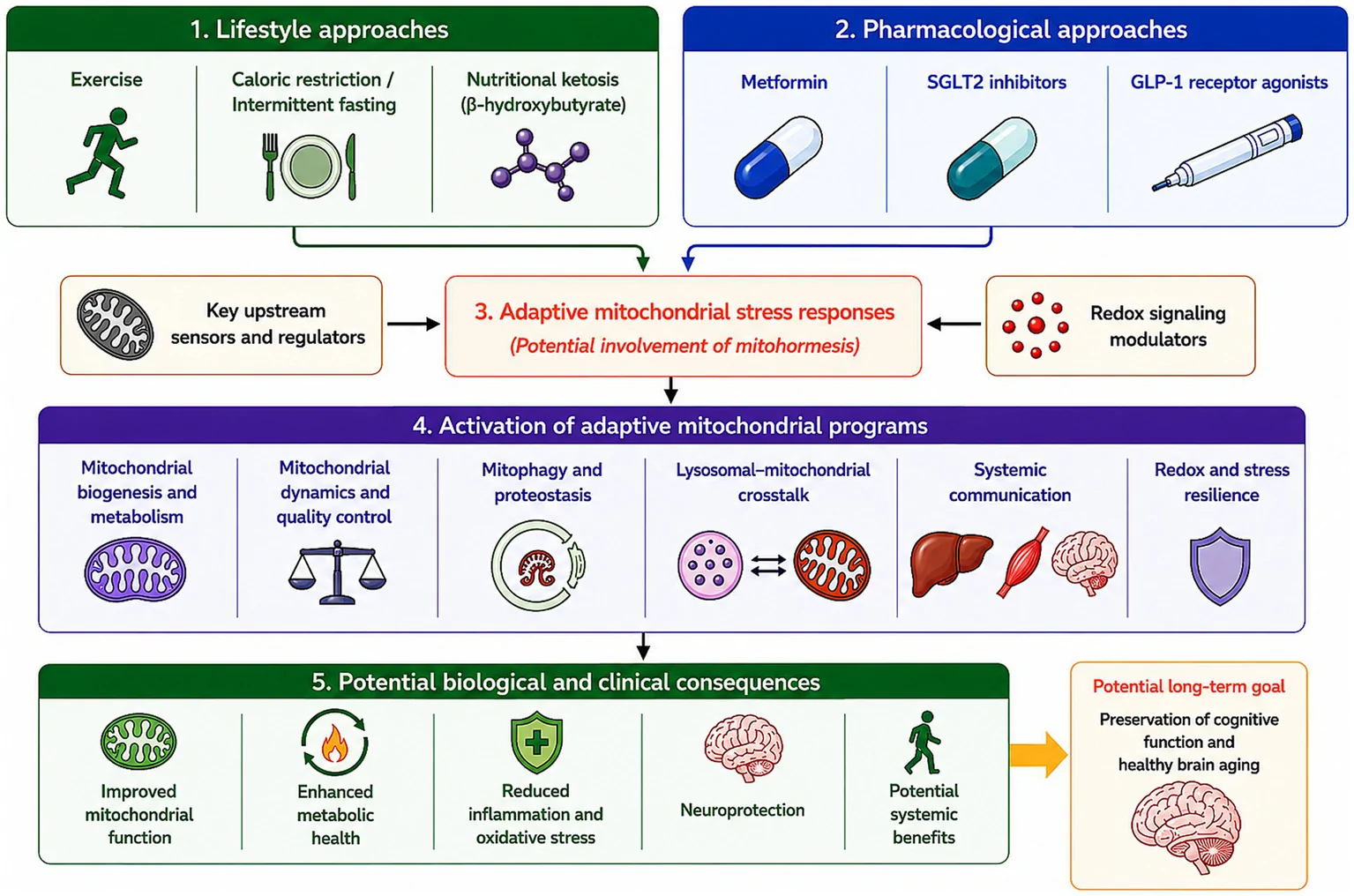
Lifestyle and pharmacological modulation of adaptive mitochondrial stress responses and their potential biological and clinical consequences. Lifestyle and pharmacological interventions, including exercise, caloric restriction, nutritional ketosis, metformin, SGLT2 inhibitors, and GLP-1RAs, can modulate metabolic and mitochondrial stress-response pathways through distinct upstream mechanisms. These interventions have been associated with activation of adaptive pathways involving AMPK, NRF2, mitochondrial biogenesis, mitophagy, lysosomal–mitochondrial communication, and intercellular or inter-organ signaling mediated by EVs, mitokines, and neural pathways. These responses may contribute to improved mitochondrial and metabolic function, reduced oxidative and inflammatory stress, and enhanced cellular and neuronal resilience. However, the extent to which these beneficial effects are directly mediated by mitohormesis varies among interventions and remains predominantly indirect or hypothesized in humans. Accordingly, preservation of cognitive function and healthy brain aging should be regarded as potential long-term therapeutic goals rather than established clinical consequences of mitohormesis. AMPK, AMP-Activated Protein Kinase; EVs, Extracellular Vesicles; GLP-1RA, Glucagon-Like Peptide-1 Receptor Agonist; NRF2, Nuclear Factor Erythroid 2-Related Factor 2; SGLT2, Sodium–Glucose Cotransporter 2.

**Table 1:** Evidence hierarchy for representative interventions proposed to engage adaptive mitochondrial stress responses and mitohormesis.

Intervention	Primary target	Proposed adaptive mitochondrial mechanism	Evidence level	Evidence for mitohormesis	Clinical relevance/limitations
Metformin	Complex I/AMPK	Mild complex I inhibition, AMPK activation, PRDX2-dependent redox signaling	Cellular/mechanistic; *C. elegans*	Direct (experimental)	Experimental evidence supports stress-induced adaptive responses; whether this mechanism mediates cognitive or neuroprotective effects in humans remains unestablished.
SGLT2 inhibitors	Systemic metabolism	Energetic/substrate shift, AMPK-related signaling, improved mitochondrial efficiency, reduced inflammation	Animal; observational human; RCT (metabolic/cardiorenal outcomes)	Indirect	Strong clinical evidence for metabolic and cardiorenal benefits, but no direct evidence that mitohormesis mediates prevention of cognitive decline or dementia.
GLP-1 receptor agonists	GLP-1 receptor signaling	Reduced oxidative stress and neuroinflammation; improved mitochondrial function and quality control	Cellular; animal; observational human; RCT; clinical outcome related to AD	Hypothesized/Indirect	Strong preclinical neuroprotective evidence, but clinical mitochondrial mechanisms remain unproven; negative phase 3 evidence on AD argues against established disease-modifying neuroprotection.
Exercise	Physiological mitochondrial stress	Transient ROS production, AMPK–PGC-1α and NRF2 signaling, mitochondrial biogenesis and quality control	Mechanistic; animal; observational human; RCT	Indirect	Human cognitive and metabolic benefits are supported, but direct demonstration that mitochondrial stress is necessary for cognitive benefit remains limited.
Caloric restriction/intermittent fasting	Nutrient-sensing pathways	Energetic stress, AMPK/sirtuin signaling, autophagy/mitophagy, mitochondrial biogenesis	Cellular; animal (multiple species); limited human evidence	Indirect	Strong experimental evidence for adaptive stress responses and metabolic resilience; direct human evidence for mitohormesis-mediated dementia prevention is lacking.
Nutritional ketosis/β-hydroxybutyrate	BHB signaling	Altered substrate utilization, HDAC inhibition, FOXO3a activation, antioxidant responses	Mechanistic; animal; human nutritional studies	Indirect/Hypothesized	May improve metabolic and mitochondrial adaptation, but evidence linking mitochondrial stress-induced adaptation to clinical neuroprotection remains limited.

Evidence for mitohormesis was classified as direct, indirect, or hypothesized. Direct evidence was defined by the demonstration of a mitochondrial perturbation followed by activation of an adaptive stress response and enhanced mitochondrial or cellular resilience, ideally supported by pathway inhibition or genetic loss-of-function experiments. Indirect evidence denotes findings compatible with mitohormesis but lacking one or more of these criteria, whereas hypothesized evidence indicates that a mitohormetic mechanism remains primarily inferential.

AD, Alzheimer’s disease; AMPK, AMP-activated protein kinase; BHB, β-hydroxybutyrate; *C. elegans*, *Caenorhabditis elegans*; FOXO3a, forkhead box O3a; GLP-1RA, glucagon-like peptide-1 receptor agonist; HDAC, histone deacetylase; NRF2, nuclear factor erythroid 2-related factor 2; PGC-1α, peroxisome proliferator-activated receptor γ coactivator-1α; PRDX2, peroxiredoxin 2; RCT, randomized controlled trial; ROS, reactive oxygen species.

Experimental studies provide a proof of principle that pharmacological perturbations of mitochondrial metabolism can elicit adaptive stress responses and neuroprotective phenotypes under specific experimental conditions (Teramayi et al., 2024). Such findings support the therapeutic potential of controlled mitochondrial stress adaptation but should not be generalized to interventions for which a defined mitochondrial perturbation and subsequent enhancement of cellular resilience have not been demonstrated.

For conceptual purposes, the interventions discussed below can be broadly classified according to their predominant mechanisms of action: (1) direct mitochondrial modulators, such as metformin, which can perturb mitochondrial energetics and activate intracellular stress-response pathways; (2) systemic metabolic modulators, including sodium–glucose cotransporter 2 (SGLT2) inhibitors and glucagon-like peptide-1 receptor agonists (GLP-1RAs), which primarily influence mitochondrial function indirectly through changes in whole-body energy metabolism and metabolic stress; and (3) physiological interventions, including exercise, caloric restriction, fasting, and nutritional ketosis, which can generate transient metabolic and mitochondrial perturbations and engage adaptive stress responses.

Despite their distinct upstream mechanisms, these interventions can converge on pathways involved in redox signaling, mitochondrial quality control, metabolic reprogramming, and stress-responsive transcriptional regulation (Sun et al., 2024). Adaptive responses may also extend beyond individual cells through inter-organ signaling mechanisms, including extracellular vesicle-mediated communication (Crewe et al., 2021). However, convergence on these pathways alone does not establish mitohormesis. The strength of evidence for a mitohormetic mechanism depends on whether a defined mitochondrial perturbation is followed by an adaptive response and demonstrable enhancement of cellular or organismal resilience.

### 5.2 Metformin and direct mitochondrial modulation

Metformin represents one of the direct experimental examples of pharmacological modulation of mitochondrial stress responses. Through partial inhibition of mitochondrial complex I, metformin can induce mild energetic and redox perturbations that activate AMPK and other adaptive stress-response pathways. In *Caenorhabditis elegans* and isolated mitochondrial preparations, metformin increased mitochondrial ROS production and activated peroxiredoxin 2 (PRDX2)-dependent signaling, resulting in enhanced stress resistance and longevity (De Haes et al., 2014). Because these experiments demonstrate a defined mitochondrial perturbation followed by activation of an adaptive stress response and enhanced resilience phenotype, they provide relatively direct experimental evidence consistent with mitohormesis.

However, whether the clinical benefits of metformin in humans are mediated by the same mitohormetic mechanism remains uncertain. Metformin exerts multiple systemic metabolic effects, and direct evidence in humans demonstrating a defined mitochondrial perturbation followed by adaptive signaling and subsequent enhancement of cellular or organismal resilience remains limited. Thus, the evidence for metformin-induced mitohormesis is mechanistically stronger in experimental systems than in clinical settings.

### 5.3 SGLT2 inhibitors and systemic metabolic adaptation

Among pharmacological interventions, SGLT2 inhibitors, including empagliflozin, dapagliflozin, and canagliflozin, have demonstrated robust cardiovascular and renal protective effects in patients with type 2 diabetes mellitus (Zinman et al., 2015; Neal et al., 2017; Wiviott et al., 2019). Beyond these established benefits, accumulating observational evidence suggests that SGLT2 inhibitor use may also be associated with a low risk of dementia. A multicenter retrospective cohort study using electronic health record databases from 11 Korean hospitals reported that SGLT2 inhibitor therapy was associated with significantly lower risks of all-cause dementia and AD than treatment with thiazolidinediones, including pioglitazone, rosiglitazone, and lobeglitazone (Park et al., 2026). In addition, a recent systematic review and meta-analysis of nine retrospective cohort studies involving more than 2.4 million patients reported that SGLT2 inhibitor therapy was associated with lower risks of all-cause dementia, AD, and vascular dementia compared with DPP-4 inhibitors, with particularly favorable associations observed for empagliflozin and dapagliflozin (Kumari et al., 2026).

Experimental and clinical studies further suggest that SGLT2 inhibitors can induce systemic metabolic adaptations associated with improved mitochondrial bioenergetics, reduced oxidative and inflammatory stress, and activation of adaptive mitochondrial stress-response pathways. In our previous prospective multicenter study involving 123 patients with type 2 diabetes, 4 months of SGLT2 inhibitor therapy significantly reduced albuminuria together with circulating tumor necrosis factor receptor (TNFR) 1 and TNFR2 levels. Notably, reductions in TNFR1 were independently associated with improvements in albuminuria, supporting anti-inflammatory effects beyond glucose lowering (Otoda et al., 2024).

Consistent with these findings, experimental and clinical evidence indicates that SGLT2 inhibitors can modulate inflammatory signaling, mitochondrial function, and cellular stress responses (Otoda et al., 2025b, 2025c). Furthermore, in our recent 24-week randomized controlled trial involving 76 participants with type 2 diabetes (median age, 66 years), empagliflozin improved vascular, hepatic, and cardiac stress markers, including cardio-ankle vascular index, fibrosis-4 index, and N-terminal pro-B-type natriuretic peptide levels, whereas both empagliflozin and imeglimin increased antioxidant capacity and reduced circulating TNFR1 and TNFR2 levels (Otoda et al., 2025c). However, cognitive outcomes, defined mitochondrial perturbations, and direct biomarkers of mitohormesis were not evaluated. Therefore, these findings support systemic metabolic and stress adaptation but provide only indirect clinical evidence for a mitohormetic mechanism.

Accordingly, SGLT2 inhibitors should not currently be regarded as established inducers of mitohormesis in humans. Their cardiovascular, renal, and potential neuroprotective effects may involve pathways that overlap with adaptive mitochondrial stress responses, but whether mitohormesis directly mediates these clinical benefits remains to be established.

### 5.4 GLP-1 receptor agonists and neuroprotective signaling

GLP-1RAs, including liraglutide, semaglutide, dulaglutide, and exenatide, have emerged as key therapeutic agents with pleiotropic effects that extend beyond glycemic control. In addition to improving metabolic homeostasis, GLP-1RAs have shown to modulate mitochondrial function, reduce oxidative stress, attenuate systemic inflammation, and exert neuroprotective effects in experimental models.

Studies in APP/PS1 transgenic mice, a widely used mouse model of AD, reported that semaglutide reduced amyloid-β (Aβ) plaque deposition in the brain, suppressed microglial and astrocyte activation, decreased inflammatory cytokine production, and improved spatial learning and memory as assessed using the Morris water maze test (Zhai et al., 2025). Histopathological analyses, immunofluorescence staining, enzyme-linked immunosorbent assay, transcriptomic profiling, and western blotting further demonstrated that semaglutide activated AMPK and inhibited Toll-like receptor 4/nuclear factor kappa B signaling, thereby attenuating neuroinflammation in this experimental model (Zhai et al., 2025). These findings suggest that GLP-1 receptor agonists may promote neuroprotection through modulation of mitochondrial stress responses and neuroinflammatory signaling. This interpretation was further supported by a subsequent study in APP/PS1 transgenic mice, which reported that semaglutide ameliorated cognitive impairment, reduced Aβ deposition, and suppressed neuroinflammation through modulation of AMPK-dependent and inflammatory signaling pathways (Yuan et al., 2026).

However, these robust preclinical findings have not translated into demonstrable disease-modifying efficacy in patients with AD. The evoke and evoke+ trials were two large, randomized, double-blind, placebo-controlled phase 3 trials evaluating oral semaglutide in 3,808 participants with amyloid-confirmed early symptomatic AD. At 104 weeks, semaglutide did not significantly reduce clinical disease progression compared with placebo, as assessed using the Clinical Dementia Rating–Sum of Boxes score in either trial (Cummings et al., 2026). These findings are consistent with the broader heterogeneity of clinical evidence regarding the cognitive effects of GLP-1RAs (Choudhury et al., 2026) and demonstrate that improvements in mitochondrial, metabolic, and neuroinflammatory pathways observed in experimental models do not necessarily translate into clinically meaningful slowing of AD progression.

Within the framework adopted in this review, GLP-1 receptor agonists should not currently be considered established inducers of mitohormesis in humans. Rather, their potential mitohormetic effects remain indirect or hypothesized because GLP-1RAs primarily improve metabolic homeostasis and attenuate oxidative and inflammatory stress, while direct evidence demonstrating a defined mitochondrial perturbation followed by adaptive signaling and enhanced cellular resilience is limited. Thus, although GLP-1RAs may preserve mitochondrial function and neuronal resilience under metabolic stress, the negative evoke and evoke+ results emphasize an important translational gap between mechanistic or preclinical evidence and clinical neurodegenerative outcomes.

### 5.5 Physiological modulation of adaptive mitochondrial stress responses

Exercise represents one of the best-characterized physiological contexts in which transient metabolic and mitochondrial stress is followed by adaptive cellular responses. Acute exercise increases energetic demand and can generate transient increases in mitochondrial ROS, which function as signaling cues that activate adaptive pathways including AMPK and NRF2. AMPK promotes mitochondrial biogenesis through peroxisome proliferator-activated receptor γ coactivator-1α (PGC-1α) and enhances fatty acid oxidation and metabolic flexibility, whereas NRF2 activates antioxidant and mitochondrial quality-control programs. These coordinated responses are consistent with the mechanistic framework of physiological mitohormesis, particularly when transient metabolic or mitochondrial perturbation is followed by enhanced stress resistance and metabolic resilience (Hawley et al., 2014; Sun et al., 2024).

Exercise is also associated with structural and functional benefits in the brain, although these observations do not by themselves establish mitohormesis as the underlying mechanism. In a randomized controlled trial involving older adults, aerobic exercise increased hippocampal volume and improved memory performance (Erickson et al., 2011). Experimental studies summarized in recent reviews, including APP/PS1 and 5 × FAD mouse models of AD and complementary neuronal cell models, have further reported that regular aerobic exercise enhances autophagy–lysosomal function, promotes clearance of amyloid-β (Aβ) and hyperphosphorylated tau, improves hippocampal synaptic plasticity, and preserves cognitive function (Li et al., 2026). Thus, exercise provides relatively strong mechanistic evidence consistent with physiological mitohormesis in experimental systems, whereas evidence that mitohormesis directly mediates its cognitive benefits in humans remains indirect.

Caloric restriction and intermittent fasting represent additional physiological interventions that engage evolutionarily conserved nutrient- and energy-sensing pathways. Experimental studies in yeast, *Caenorhabditis elegans*, *Drosophila*, and rodent models have reported activation of AMPK and sirtuin signaling, stimulation of mitochondrial biogenesis and autophagy, improvements in mitochondrial efficiency and metabolic flexibility, and increased cellular stress resistance (Madeo et al., 2019). These findings are compatible with mitohormetic adaptation in experimental systems. However, activation of nutrient-sensing and mitochondrial quality-control pathways alone is not sufficient to establish mitohormesis, and direct evidence demonstrating that a defined mitochondrial perturbation mediates the long-term benefits of caloric restriction or intermittent fasting in humans remains limited.

Nutritional ketosis may also engage adaptive mitochondrial and redox responses consistent with mitohormesis. In rodent models, ketogenic diets have been shown to produce a temporal sequence characterized by an early increase in mitochondrial ROS and oxidative stress, followed by activation of NRF2-dependent antioxidant signaling and a subsequent reduction in mitochondrial ROS below baseline levels, providing relatively direct experimental evidence of a mitohormetic response (Miller et al., 2018). In humans, ketogenic diets and fasting increase circulating β-hydroxybutyrate (BHB), but direct demonstration of a comparable temporal mitohormetic response remains limited. BHB may additionally promote cellular stress resistance through signaling mechanisms that overlap with, but are not necessarily equivalent to, mitohormesis. Experimental studies have shown that BHB inhibits class I and II histone deacetylases (HDACs), enhances forkhead box O3a-mediated antioxidant defenses, including superoxide dismutase 2 and catalase, and improves resistance to oxidative stress (Miller et al., 2018). Thus, nutritional ketosis provides relatively strong mechanistic evidence consistent with mitohormesis in experimental models, whereas evidence that the same mechanism mediates its physiological or clinical effects in humans remains indirect.

### 5.6 Disease-modifying therapies and upstream mitochondrial adaptation

Recent advances in disease-modifying therapies for AD, including lecanemab and donanemab, have focused on monoclonal antibodies directed against Aβ (Mintun et al., 2021; van Dyck et al., 2023). Concurrently, emerging therapeutic strategies targeting intracellular protein-clearance pathways have yielded encouraging preclinical findings. Experimental studies in mouse models of tauopathy have reported that engineered single-domain antibodies facilitate tau degradation through the autophagy–lysosomal pathway, thereby reducing tau accumulation and improving neuronal function (Jiang et al., 2026).

These therapeutic approaches target disease-associated protein pathology, including Aβ deposition and tau aggregation. In parallel, mitochondrial dysfunction, impaired proteostasis, oxidative stress, and metabolic dysregulation are increasingly recognized as interconnected processes that may contribute to neuronal vulnerability and interact bidirectionally with established AD pathology. Accordingly, strategies that preserve mitochondrial quality control and adaptive stress responses could potentially complement Aβ- and tau-directed therapies by supporting neuronal resilience rather than by directly targeting protein aggregation. Within this framework, interventions capable of modulating adaptive mitochondrial stress responses may provide an additional therapeutic dimension, although direct evidence that mitohormesis mediates clinically meaningful neuroprotection in humans remains limited.

Thus, protein-directed disease-modifying therapies and approaches targeting mitochondrial stress adaptation should be viewed as potentially complementary rather than as interventions acting at strictly defined downstream and upstream stages of a linear pathogenic cascade. Determining whether modulation of mitochondrial stress responses can enhance or extend the clinical benefits of established disease-modifying therapies will require prospective experimental and clinical investigation.

### 5.7 Future therapeutic perspectives

The studies discussed above indicate that pharmacological and lifestyle interventions can converge on adaptive pathways involved in mitochondrial quality control, redox homeostasis, metabolic reprogramming, and inter-organ communication, despite acting through distinct upstream mechanisms (Cheng et al., 2023; Sun et al., 2024; Gunawan et al., 2025). These shared responses provide a conceptual framework for understanding how modulation of mitochondrial stress adaptation may contribute to the pleiotropic effects of diverse interventions. However, convergence on common adaptive pathways should not itself be interpreted as evidence that mitohormesis directly mediates their beneficial effects.

Future therapeutic strategies may therefore benefit from combining approaches directed at established disease-associated pathology with interventions that preserve mitochondrial quality control, proteostasis, and organelle communication before adaptive capacity is irreversibly compromised. Such approaches may be relevant to both metabolic and neurodegenerative diseases, although their therapeutic effects are likely to depend on disease stage, tissue context, stress intensity, and the timing of intervention. Although direct clinical evidence for mitohormesis remains limited, metabolic therapies have been reported to improve mitochondrial function, redox homeostasis, and inflammatory signaling in humans (Otoda et al., 2025b, 2025c). Whether these effects represent mitohormesis and whether they translate into clinically meaningful preservation of neuronal or cognitive function remain to be established.

The principal molecular targets, levels of evidence, strength of evidence for mitohormesis, and potential clinical implications of representative interventions are summarized in Table 1 and integrated conceptually in Figure 3. Future studies should establish whether defined mitochondrial perturbations are followed by adaptive responses and demonstrable enhancement of cellular, tissue, or organismal resilience, while also defining dose–response relationships, temporal dynamics, tissue specificity, and clinically relevant biomarkers.

## 6 Translational barriers and unresolved questions

Despite growing experimental evidence supporting adaptive mitochondrial stress responses, several major barriers currently limit the translation of mitohormesis into clinical strategies for metabolic and neurodegenerative diseases. A fundamental challenge is the lack of validated biomarkers that can distinguish beneficial mitohormetic responses from nonspecific mitochondrial stress or progressive mitochondrial dysfunction in humans. Circulating stress-responsive factors, including FGF21 and GDF15, as well as changes in redox biomarkers and mitochondrial metabolites, may provide information on mitochondrial stress, but none currently represents a specific biomarker of mitohormesis. Moreover, activation of AMPK, NRF2, mitophagy, or other adaptive pathways alone does not establish mitohormesis unless the initial mitochondrial perturbation can be linked to a subsequent increase in cellular or tissue resilience.

A second challenge is the context- and dose-dependent nature of mitohormesis. The biological consequences of mitochondrial stress are influenced by its intensity, duration, and frequency, as well as by age, metabolic status, genetic background, and disease stage. A mitochondrial perturbation that elicits an adaptive response in healthy or early-stage disease may exceed the adaptive capacity of cells in advanced metabolic or neurodegenerative disease. Defining the therapeutic window in which mitochondrial stress remains adaptive rather than maladaptive will therefore be essential for the development of interventions based on mitohormetic principles.

Tissue specificity represents an additional translational barrier. Mitochondrial responses measured in circulating cells, skeletal muscle, liver, or other peripheral tissues may not directly reflect mitochondrial stress adaptation in neurons or glial cells. The blood–brain barrier and the metabolic specialization of different neural cell populations further complicate extrapolation from systemic biomarkers to the central nervous system. In addition, dementia represents a heterogeneous group of disorders with distinct and overlapping contributions from amyloid and tau pathology, cerebrovascular disease, inflammation, and metabolic dysfunction. Consequently, the contribution of impaired mitochondrial stress adaptation is unlikely to be uniform across patients or disease subtypes.

Finally, substantial gaps remain between mechanistic, preclinical, and clinical evidence. Many interventions discussed in this review improve mitochondrial function, redox homeostasis, or inflammatory signaling, but these effects do not necessarily demonstrate that mitohormesis mediates their clinical benefits. The negative phase 3 results of semaglutide in early symptomatic AD, despite encouraging experimental evidence, illustrate this translational gap. Future studies should therefore incorporate longitudinal assessment of mitochondrial stress and adaptation, define dose–response and temporal relationships, and, where feasible, establish mechanistic links between adaptive mitochondrial signaling and clinically meaningful outcomes. Such approaches will be necessary to determine whether mitohormesis can be translated from a compelling biological framework into a measurable and therapeutically actionable process in humans.

## 7 Conclusion

Mitohormesis provides a useful framework for understanding how the biological consequences of mitochondrial stress depend on its intensity, duration, and the capacity of cells to mount adaptive responses. Moderate and transient mitochondrial perturbations can activate adaptive signaling pathways that enhance mitochondrial quality control, redox homeostasis, proteostasis, and cellular resilience, whereas persistent or excessive stress can overwhelm these protective mechanisms and promote mitochondrial dysfunction and tissue injury. In metabolic diseases, chronic hyperglycemia, insulin resistance, and lipid overload may progressively shift this balance from adaptive mitochondrial stress responses toward maladaptive mitochondrial dysfunction.

These processes are particularly relevant to the central nervous system, where neuronal survival and synaptic function depend strongly on mitochondrial integrity. Evidence from experimental models and human studies supports an association between metabolic dysfunction, impaired mitochondrial homeostasis, and increased vulnerability to cognitive decline and neurodegeneration. In AD, reciprocal interactions between mitochondrial dysfunction and Aβ/tau pathology may further amplify neuronal injury, illustrating how impaired mitochondrial stress adaptation can interact with disease-specific pathology rather than acting through a strictly linear pathway.

Within this framework, mitohormesis may represent one component of the adaptive interface linking systemic metabolic stress to neuronal resilience. However, activation of AMPK, NRF2, mitophagy, mitochondrial biogenesis, or other stress-response pathways alone should not be considered sufficient evidence of mitohormesis. Although exercise, caloric restriction, nutritional ketosis, and the use of metformin, SGLT2 inhibitors, and GLP-1 receptor agonists have been associated with mitochondrial and metabolic adaptations, evidence that mitohormesis directly mediates their beneficial effects varies substantially across interventions and remains predominantly indirect or hypothesized in humans. The discrepancy between encouraging mechanistic and preclinical findings and clinical outcomes, including the lack of disease-modifying efficacy of semaglutide in recent phase 3 trials of early symptomatic AD, further emphasizes this translational gap.

Future studies should therefore move beyond documenting activation of adaptive mitochondrial pathways and determine whether defined mitochondrial perturbations produce subsequent increases in cellular, tissue, or organismal resilience. The development of validated biomarkers, characterization of dose–response and temporal relationships, consideration of tissue and disease specificity, and demonstration of clinically meaningful outcomes will be essential for establishing the relevance of mitohormesis in humans.

Collectively, current evidence supports mitohormesis as a conceptual framework for integrating mitochondrial stress adaptation, metabolic dysfunction, and neuronal resilience, rather than as an established unifying mechanism of neurodegeneration. Defining when mitochondrial stress is adaptive, when adaptive capacity becomes insufficient, and whether these responses can be safely and effectively modulated in humans will be critical for determining the therapeutic potential of mitohormesis in metabolic and neurodegenerative diseases.

## Glossary

Aβ: amyloid-β
AD: Alzheimer’s disease
AMPK: AMP-activated protein kinase
ATP: adenosine triphosphate
ATF4: activating transcription factor 4
BHB: β-hydroxybutyrate
*C. elegans*: *Caenorhabditis elegans*
EV: extracellular vesicle
FGF21: fibroblast growth factor 21
GDF15: growth differentiation factor 15
GLP-1RA: glucagon-like peptide-1 receptor agonist
HRI: heme-regulated inhibitor kinase
ISR: integrated stress response
MRI: magnetic resonance imaging
mTORC1: mechanistic target of rapamycin complex 1
mtDNA: mitochondrial DNA
NRF2: nuclear factor erythroid 2-related factor 2
OMA1: overlapping activity with m-AAA protease 1
OXPHOS: oxidative phosphorylation
Parkin: Parkin E3 ubiquitin protein ligase
PGC-1α: peroxisome proliferator-activated receptor γ coactivator-1α
PINK1: PTEN-induced kinase 1
POMC: proopiomelanocortin
ROS: reactive oxygen species
SGLT2: sodium–glucose cotransporter 2
TFAM: mitochondrial transcription factor A
TNFR: tumor necrosis factor receptor
UPR*^mt^*: mitochondrial unfolded protein response.
